# International medical graduates: how can UK psychiatry do better?

**DOI:** 10.1192/bjb.2020.118

**Published:** 2021-10

**Authors:** Emmeline Lagunes-Cordoba, Raka Maitra, Subodh Dave, Shevonne Matheiken, Femi Oyebode, Jean O'Hara, Derek K. Tracy

**Affiliations:** 1 Camden and Islington NHS Foundation Trust, UK; 2 Tavistock and Portman NHS Foundation Trust, UK; 3 Derbyshire Healthcare NHS Foundation Trust, UK; 4 East London NHS Foundation Trust, UK; 5 National Centre for Mental Health, UK; 6 South London and Maudsley NHS Foundation Trust, UK; 7 Oxleas NHS Foundation Trust, UK

**Keywords:** IMGs, NHS, BAME, career, stigma and discrimination

## Abstract

The National Health Service (NHS) was created 70 years ago to provide universal healthcare to the UK, and over the years it has relied upon international medical graduates (IMGs) to be able to meet its needs. Despite the benefits these professionals bring to the NHS, they often face barriers that hinder their well-being and performance. In this editorial, we discuss some of the most common challenges and the adverse effects these have on IMGs’ lives and careers. However, we also propose practical measures to improve IMGs’ experiences of working in psychiatry.

The staff of the National Health Service (NHS) includes many doctors who have trained abroad. Unfortunately, despite their contributions, many international medical graduates (IMGs) face considerably greater difficulties than UK graduates. Longitudinal data clearly show differential attainment of IMG doctors in both postgraduate examinations and more senior clinical, academic and managerial positions.^1,2^ They are also more likely to be reported to the General Medical Council (GMC) for misconduct and to have such complaints upheld.^3^ This is detrimental to their well-being and risks affecting the quality of care provided by them.

There are many contributing factors, from direct discrimination, through a lack of familiarity with and support from the UK system, to a failure to harness IMGs’ strengths. This editorial will explore this, taking the available evidence and experiences of the authors to propose positive next steps for individuals and organisations.

## IMGs: who are they?

### Definitions of IMGs: a heterogeneous group

The GMC defines an IMG as someone who has obtained their primary medical qualification outside the European Economic Area (EEA).^4^ However, that simple definition covers a range of complexities. It can encompass a childhood in another culture and different intersectional experiences of nationality, religion, gender and skin colour. It involves medicine studied in a different healthcare system, with nuanced variations in communication and therapeutic relationships. Some things, however, are common to most IMGs: personal and professional loss from the country they left; a need to build a network of friends and embrace a new life; exposure to a new environment and health system; and the hope for a better future, which despite any adversity keeps many motivated to continue. However, every IMG's journey is unique, and the challenges faced will depend on the interplay of many factors; for example, we note that technically the term ‘IMG’ applies to a White British citizen who studies abroad and returns to work in the UK, yet such an individual is less likely to face attainment gaps.

There is no single route for an IMG to transition into the NHS. Those from an EEA country or Switzerland (not considered IMGs by the GMC) are eligible for full GMC registration and licence to practise medicine in the UK as long as they demonstrate proficiency in English. For other IMGs, the most common method of obtaining registration is by passing the Professional and Linguistic Assessments Board (PLAB) examination,^5^ an initiative designed to ensure parity of medical education and training standards. Post-PLAB doctors are then free to apply to appropriate training schemes, and to work as specialty and associate specialist (SAS) or locally employed doctors. Another route is the Medical Training Initiative (MTI) scheme sponsored by a UK Medical Royal College, faculty or GMC-approved institution for postgraduate training. Some IMGs can also gain registration if they hold a relevant postgraduate qualification from an approved overseas awarding body.^6^ Finally, IMGs can get their registration by applying for a Certificate of Eligibility for Specialist Registration. This requires doctors to demonstrate that their training matches the UK equivalent.^7^

However, once an IMG starts working in the NHS, they are expected to adhere to the norms outlined in the GMC's *Good Medical Practice* guide.^8^ Some trusts invest in providing appropriate induction and extra support; however, there is currently no clear national guidance or requirement for either organisations or IMGs on how to support this transition into the UK.

### Data on IMG numbers and specialties/roles

A report from the House of Commons noted that of doctors in non-primary care settings, 13% are from Asia, 9.1% are from the European Union and 4.8% are from Africa.^9^ IMGs account for 60% of non-consultant and non-training doctors currently working in the UK.^10^ It is not clear why IMGs are more likely to work as SAS or locally employed doctors. Some might have found these roles more suitable to gain experience before enrolling in formal training, while some might be less interested in the recognised challenges that come with traditional training and consultant roles. However, some people may prefer a more flexible role for reasons including childcare, especially as they might have less family and informal support than British doctors; while others might find it practically easier to obtain such a job. Having said this, it is also likely that for a fair few it is a second-choice career pathway on account of failure to succeed in passing the relevant postgraduate examinations. Having a non-UK primary medical qualification has the largest influence on postgraduate examination attainment; the pass rate in some postgraduate examinations is 35% for IMGs, compared with 80% for UK graduates.^11^ These attainment differences have also been identified between IMGs and UK graduate doctors training in psychiatry.^12^

Psychiatry is particularly popular among IMGs,^12^ who account for 44% of psychiatry trainees.^10^ A preference for psychiatry has also been demonstrated in the USA^13^ and among UK doctors from Black and minority ethnic (BAME) backgrounds.^14^ The reason for this is not clear, although psychiatry has long had a particular recruitment problem,^15^ and it is possible that a need to pass the residence labour market test may be encouraging some to take posts that are more easily available. The authors’ own discussions include the reflection that many IMGs may come from cultures where mind and body are not so separated, and find a natural affinity with psychiatry.

## IMGs: challenges they face

### Practical and cultural aspects to change

Initially, many IMGs need to find a place to live, to become orientated with public transport and potentially to learn to drive on the left side of the road. They need to open a bank account, get a UK telephone and identify where and how to access shops, sport clubs, religious centres and schools. They must adapt to the UK's many regional accents, and learn both local idioms and British colloquialisms. In addition, the first few years are often clouded by financial and visa-related stresses.

IMGs go through adaptation (changes in individuals or groups due to environmental demands) and acculturation (cultural changes resulting from encounters with members of different groups).^16^ This includes learning appropriate new behaviours and unlearning behaviours that are no longer appropriate. Refugee doctors merit additional comment. They are also IMGs, but they have had to flee their home countries and lives to escape persecution or other threats. So they often have experienced trauma, more financial difficulties and less certainty regarding visas, leading them to face greater isolation. Cohn et al^17^ noted that owing to having to leave their homes rapidly and in fear, refugee doctors might not have all the paperwork required for registration, or may not be able to contact people in their home countries to get relevant documents, which may complicate their ability to fulfil GMC requirements.

### Loss of status, discrimination and racism

IMGs often not only leave behind family and friends, they almost always leave a social status, identity and trust that they previously earned and enjoyed. Many take up posts of lower grade or status than expected or warranted by their expertise.^18,19^ IMGs have reported a loss of autonomy in their decision-making,^20^ and a professional devaluation which can lead to a loss of their confidence to perform or even defend themselves in cases of harassment or where their practice is being scrutinised.^19^

Many IMGs still suffer greater levels of direct and indirect discrimination and harassment. IMGs have reported feeling discriminated against by colleagues and at an institutional level.^20^ Institutional racism has also been evidenced by bias regarding hiring practices of doctors with foreign names.^21^ Subjective bias due to racial discrimination has also been considered a factor associated with the failure of the clinical skills assessment for UK BAME and IMG doctors.^22^ A third of SAS and locally employed doctors in the UK, who are predominately IMGs, have reported experienced bullying or harassment in their workplaces, mainly characterised by rudeness.^23^

### Greater rates of complaints

The GMC's *Fair to Refer* report^3^ shows a disproportionate number of fitness to practise referrals for overseas doctors (2.5 times greater) and doctors from BAME backgrounds (two times greater) compared with White UK graduates, and they are more likely to face harsher sanctions. It is less clear whether this is compounded in IMGs from non-White backgrounds. The report offers potential explanations, including inadequate induction and support, lack of honest and effective feedback, working patterns or contracts that lead to isolation, pervasive insider–outsider dynamics, and a lack of confidence in raising concerns or challenging accusations. This last factor fits with the finding of Jalal et al that IMGs are less likely than UK graduates to report bullying.^24^ The GMC report has been criticised for not addressing why the GMC tends to give harsher sanctions to referred IMGs and BAME doctors.^25^

## Existing evidence and initiatives

### IMGs’ perspectives

Wolf et al^26^ found that IMGs and BAME trainees reported more difficulties, cultural differences and lack of trust with senior doctors, as well as biased assessments and recruitment processes. Hashim^27^ identified challenges for IMGs in understanding the NHS system and values, learning environments (with a lack of direct guidance), receiving feedback and feelings of being undervalued by colleagues. A survey of IMGs’ understanding of the GMC regulatory framework found that many were surprised or confused by the range and specificity of the regulations, including the emphasis on individual patient autonomy.^28^ It also identified that IMGs could have difficulties with nuances of non-verbal communication and UK social and behavioural norms. A key aspect was the lack of relevant information prior to registration, variable levels of training and support, and isolation in non-training posts.

### International literature on interventions

There is a limited evidence base to support interventions, including international examples from Canada,^29^ the USA,^20^ and South Africa.^30^ Two recent literature reviews^18,24^ summarised the following recommendations: individual assessments prior to induction with follow-up; making relevant information available; providing comprehensive information about the role and job; addressing the culture gap (providing supervisors and mentors, understanding clinical and cultural differences); considering different learning styles; buddy systems (for guidance and help); improving cultural awareness from the organisation; and establishing a national induction programme, complementary to local inductions. With regard to refugee doctors, Butt et al (2019) showed that formal support to gain their licence to practise was an effective intervention. Although all these could be potentially effective interventions to improve IMGs’ well-being, there still seems to be no consensus regarding which organisations are responsible for implementing them or overseeing these changes.

### Work by UK professional bodies

Some GMC initiatives have been put in place to address the differential attainment; these include the optional ‘Welcome to UK Practice’ workshop,^31^ which according to a recent report^32^ is highly valued by doctors and their supervisors, improving knowledge on ethical issues, GMC guidance and UK practice in general, as well as communication and a focus on patient centredness. It provided opportunities to meet colleagues, share learning and gain support, but the report also suggested that IMGs feel a general lack of support once they are in practice.

The MTI by the Association of Medical Royal Colleges^33^ is specifically for IMGs and provides a helpful guide^34^ to aid with preparation for relocation, as well as providing mentorship on starting the scheme. A recent evaluation of the MTI psychiatry scheme reported that enrolled IMGs highly rated their clinical supervision and overall experience with this initiative.^35^ The British Medical Association (BMA) website also has a section focusing on IMGs, including information regarding life and work in the UK;^36^ while we were preparing this manuscript, the BMA sent their very first IMG newsletter, focused on key news and information relevant to IMGs. Online communities are mushrooming to support IMGs while they redefine their identities in a new culture with its evident uniqueness.

This differential IMG attainment also affects membership and representation within the Royal Colleges, as SAS doctors do not have the same rights as fully registered members of some Royal Colleges. Positively, we note that the roles and representation of SAS doctors within the Royal College of Psychiatrists is currently under review. The Royal College of Psychiatrists has a trainee support group which provides guidance regarding the needs of IMG trainees to heads of schools of psychiatry across the UK to improve differential attainment, while the Psychiatric Trainee Committee is currently drafting a guide that will contain sections to support IMGs, including cultural induction to the UK, training pathways, exams and visa information. The college also organises workshops to help supervisors to gain the knowledge and skills to mentor and supervise an IMG doctor, and conferences to foster networking among IMGs. We also recognise that the College has recently established two new professional leads on race equality.

## Next steps

### Recognising the wider societal issues: Black Lives Matter and Covid-19

The Black Lives Matter movement has re-emphasised the social disparities between White British people and those of BAME and immigrant backgrounds. It reminds us that many of the adverse experiences of IMGs are also encountered by UK BAME medical graduates, notably, discrimination, racism and differential attainment. Compared with their White counterparts, UK BAME doctors underperform academically;^35^ are more likely to fail their clinical skills assessments;^22^ are less likely to be accepted into a specialty training programme, apply for consultant posts, or be shortlisted for and offered consultant posts; and earn less.^37^ Wolf et al^38^ found that perceived difficulty in talking about race with trainees and isolation or non-specificity of interventions were the main barriers to improvement. The Covid-19 pandemic has also shown the different vulnerabilities of BAME staff and patients, many of which are due to fundamental societal inequities, and the need for appropriate support and care.^39^ As a positive note, it was heartening to see the *BMJ* recently commit a whole issue to the topic of racism.^40^

### Recognising IMGs’ strengths: the ‘problem’ is not in the individual

Most discussion still unconsciously locates the ‘problem’ in the IMG rather than adopting a systemic perspective and working to embrace IMGs’ strengths and their knowledge and experience of working in other socioeconomic and healthcare systems.

Many IMGs not only achieve their goals but also go on to subsequently become prominent and respected doctors, leaders in their field and admired by their peers. Within psychiatry, we have many such examples who have succeeded and developed internationally high-profile careers as clinicians and academics; the simplest scan of UK psychiatry's output and reputation will show its gains from many IMGs. The strengths of an internationally trained, multicultural and multilingual workforce need to be harnessed. Box 1 summarises our recommendations for positive change, based on an assimilation of the existing literature, our experiences as IMGs (all but one author) and practising clinicians in the UK, and our application to UK psychiatry. We have provided a suggested clustering of which organisations and individuals might be best placed to provide these.

Box 1 Recommendations for positive change, clustered by provider.

**Table:** 

Regulatory bodies
Continued evaluation and addressing the disproportionality of GMC (and local) complaints and investigations into IMGs. An open and transparent process of data collection and a clear accountability framework to ensure that progress, or its lack, in these areas can be monitored. Host IMG-specific resources and disseminate via NHS trust websites, including sharing of examples of good practice (for example, the MTI, while recognising that there can be specialty-specific challenges and opportunities.
The Royal College of Psychiatrists
Explicit investigation into differential attainment in the MRCPsych examinations. Explicit inclusion of IMGs in examination and curriculum design. Publishing data on IMG representation on College bodies. Have IMG-specific events, resources and examples of best practice for psychiatrists.
NHS trusts
Trusts have an IMG champion working with Human Resources to informon all IMG appointments. Trusts’ HR staff to receive training to enable them to deal competently with IMG issues. Trusts have explicit policy and action targeting racism, with open publication and updates. Local induction programme specifically for IMGs, mainly during the first 2 years of their career in the UK/NHS. Focused support and mentoring for IMGs new to the NHS. Encourage and facilitate IMGs’ interaction with UK graduates (e.g. universities host a Welcome Day for international students). Host events dedicated to local IMGs – to celebrate successful journeys and to foster a sense of community. Continuing professional development events to learn how IMGs’ experience in their home countries can contribute positively to improved patient care in the NHS.
Individual services, teams, supervisors/mentors and IMGs
Link up IMGs with appraisers at the time of starting work so that appropriate mentoring can be organised. Inclusion of modules focused on IMG issues for educational and clinical supervisors. Encourage IMGs to attend local Balint groups. Encourage IMGs to attend local academic days for trainees in specific specialities.

There is some overlap and the boundaries between these are only suggestions that might benefit from local change.

## Conclusion

Our personal experience – all but one of us are IMGs – has been that UK society is marked by a focus on fairness. This has motivated us to raise some of the issues that many IMGs are currently facing. We are grateful that British society and culture has, largely, welcomed IMGs and given us the freedom to make these comments. However, we also feel that for many local doctors, IMGs can become invisible and their specific strengths and learning needs go unnoticed. We do recognise that each IMG has had a different journey, with many factors affecting their own challenges – gender, ethnicity, skin colour, religion and sexual orientation – just like every non-IMG doctor. Motivated by personal challenges, dreams of a new life or even external factors uncontrolled by them, each IMG has to go through a process of acculturation, and loss of former identity and building of a new one, influenced by their professional and personal experiences.

For IMGs, there is much to learn personally and professionally upon coming to the UK. In healthcare, this includes familiarising oneself with the ‘process of regulation, challenging, making appeals’ and fostering a culture of ‘learning not blaming’. In society, every citizen has the responsibility to respect the rights of others and to treat others with fairness; in return, the UK offers freedom of speech and freedom from unfair discrimination.

UK healthcare systems, regulatory bodies, Royal Colleges, NHS trusts, medical leaders and indeed all of us are responsible for being aware of IMGs’ struggles, as these can have long-lasting effects not only on IMGs’ careers and lives but also on the care of the patients they serve. If we can start to demonstrate a degree of compassionate curiosity about IMGs, appreciating their diversity and strengths, the new knowledge, skills and wisdom that we will acquire can only lead to better patient care and a happier workforce.

## About the authors

**Dr Emmeline Lagunes-Cordoba,** Camden and Islington NHS Foundation Trust, and the Department of Health Service and Population Research, Institute of Psychiatry, Psychology & Neuroscience, King's College London, UK; **Dr Raka Maitra**, Tavistock and Portman NHS Foundation Trust, Trainee Support Group, Royal College of Psychiatrists, and Department of Psychosis Studies, Institute of Psychiatry, Psychology & Neuroscience, King's College London, UK; **Dr Subodh Dave**, Trainee Support Group, Royal College of Psychiatrists, and Derbyshire Healthcare NHS Foundation Trust, UK; **Dr Shevonne Matheiken**, East London NHS Foundation Trust, London, UK; **Professor Femi Oyebode**, National Centre for Mental Health, Birmingham, UK; **Dr Jean O'Hara**, South London and Maudsley NHS Foundation Trust and Department of Psychological Medicine, Institute of Psychiatry, Psychology & Neuroscience, King's College London, UK; **Dr Derek K. Tracy**, Oxleas NHS Foundation Trust, London, UK and Department of Psychosis Studies, Institute of Psychiatry, Psychology & Neuroscience, King's College London, UK. E.L.C., R.M., S.D., S.M., F.O. and D.T. identify as IMGs, and J.O.H. as a UK BAME graduate. The authors note that their own journeys have informed this piece. They further reflect that their challenges, opportunities, and the various intersectional issues they have faced have all been unique, and some have faced greater and lesser adversities than others.

## References

[1] General Medical Council. What are we Doing to Address Differential Attainment? GMC, 2020 (https://www.gmc-uk.org/education/standards-guidance-and-curricula/projects/differential-attainment/what-are-we-doing-to-address-it).

[2] Wolf K. Differential attainment in medical education and training. BMJ 2020; 368. Available from: 10.1136/bmj.m339. 32047006

[3] General Medical Council. Fair to Refer? GMC, 2019 (www.gmc-uk.org/about/what-we-do-and-why/data-and-research/research-and-insight-archive/fair-to-refer).

[4] HNS Employers. Working and Training in the National Health Service. NHS Employers, 2015.

[5] General Medical Council. Professional and Linguistic Assessments Board. GMC, 2020 (https://www.gmc-uk.org/registration-and-licensing/join-the-register/plab).

[6] Royal College of Psychiatrists. Medical Training Initiative (MTI). Royal College of Psychiatrists. 2020 (https://www.rcpsych.ac.uk/training/MTI).

[7] General Medical Council. Certificate of Eligibility for Specialist Registration or Certificate of Eligibility for GP Registration Application. GMC, 2020 (https://www.gmc-uk.org/registration-and-licensing/join-the-register/registration-applications/specialist-application-guides/specialist-registration-cesr-or-cegpr).

[8] General Medical Council. Good Medical Practice. GMC, 2019 (https://www.gmc-uk.org/ethical-guidance/ethical-guidance-for-doctors/good-medical-practice).

[9] Baker C. NHS Staff from Overseas: Statistics. House of Commons, 2020.

[10] General Medical Council. The State of Medical Education and Practice in the UK: The Workforce Report. GMC, 2019 (https://www.gmc-uk.org/-/media/documents/the-state-of-medical-education-and-practice-in-the-uk---workforce-report_pdf-80449007.pdf).

[11] General Medical Council. Specialty Exam Pass Rates for Candidates by PMQ & Ethnic Group. GMC, 2020.

[12] Fazel S, Ebmeier K. Specialty choice in UK junior doctors: is psychiatry the least popular specialty for UK and international medical graduates? BMC Med Educ 2009; 9: 77. Available from: 10.1186/1472-6920-9-77. 20034389 PMC2805648

[13] Rao N, Yager J. Acculturation, education, training, and workforce issues of IMGs: current status and future directions. Acad Psychiatry 2012; 36(4): 268–70. 22851021 10.1176/appi.ap.12050095

[14] Rodriguez Santana I, Chalkley M. Getting the right balance? A mixed logit analysis of the relationship between UK training doctors’ characteristics and their specialties using the 2013 National Training Survey. BMJ Open 2017; 7. Available from: 10.1136/bmjopen-2016-015219. PMC5724110 28801397

[15] Pandian H, Mohamedali Z, Chapman G, Vichenzo P, Ahmed S, Mulliez Z, PsychSocs: student-led psychiatry societies, an untapped resource for recruitment and reducing stigma? BJPsych Bulletin 2020; 44(3): 91–5. 31950893 10.1192/bjb.2019.88 PMC8058822

[16] Berry JW. Immigration, acculturation and adaptation. Appl Psychol 1997; 46(1): 5–68.

[17] Cohn S, Alenya J, Murray K, Bhugra D, De Guzman J, Schmidt U. Experiences and expectations of refugee doctors. BJPsych 2006; 189: 74–8. 16816309 10.1192/bjp.bp.105.010975

[18] Kehoe A, McLachlan J, Metcalf J, Forrest S, Carter M, Illing J. Supporting international medical graduates’ transition to their host-country: realist synthesis. Med Educ 2016; 50: 1015–1032. 27628719 10.1111/medu.13071 PMC5113661

[19] Wong A, Lohfeld L. Recertifying as a doctor in Canada: international medical graduates and the journey from entry to adaptation. Med Educ 2007; 42: 53–60. 18086199 10.1111/j.1365-2923.2007.02903.x

[20] Chen PG, Nunez-Smith M, Bernheim S, Berg D, Gozu A, Curry L. Professional experiences of international medical graduates practicing primary care in the United States. J Gen Intern Med 2010; 25(9): 947–53. 20502974 10.1007/s11606-010-1401-2 PMC2917670

[21] Esmail A, Everington S. Racial discrimination against doctors from ethnic minorities. BMJ Clin Res 1993; 306: 691–692. 10.1136/bmj.306.6879.691 PMC1677082 8471921

[22] Esmail A, Roberts C. Academic performance of ethnic minority candidates and discrimination in the MRCGP examinations between 2010 and 2012: analysis of data. BMJ 2013; 347. Available from: 10.1136/bmj.f5662. PMC3898419 24072882

[23] Genral Medical Council. Survey of Specialty and Associate Specialist (SAS) and Locally Employed (LE) Doctors. GMC, 2019.

[24] Jalal M, Bardhan K, Sanders D, Illing J. Overseas doctors of the NHS: migration, transition, challenges and towards resolution. Future Healthc J 2019; 6(1): 76–81. 10.7861/futurehosp.6-1-76 PMC6520089 31098591

[25] Majid A. What lies beneath: getting under the skin of GMC referrals. BMJ 2020; 368. Available from: 10.1136/bmj.m338. 32051125

[26] Woolf K, Rich A, Viney R, Needleman S, Griffin A. Perceived causes of differential attainment in UK postgraduate medical training: a national qualitative study. BMJ Open 2016; 6. Available from: 10.1136/bmjopen-2016-013429. PMC5168507 27888178

[27] Hashim A. Educational challenges faced by international medical graduates in the UK. Adv Med Educ Pract 2017; 8: 441–5. 28721119 10.2147/AMEP.S126859 PMC5498680

[28] Slowther A, Lewando Hundt G, Purkis J, Taylor R. Experiences of non-UK-qualified doctors working within the UK regulatory framework: a qualitative study. J R Soc Med 2012; 105: 157–65. 22408082 10.1258/jrsm.2011.110256 PMC3343706

[29] Kirmayer LJ, Sockalingam S, Po-Lun Fung K, Fleisher W, Adeponle A, Bhat V. International Medical Graduates in Psychiatry: Cultural Issues in Training and Continuing Professional Development. Canadian Psychiatric Association, 2018. 10.1177/0706743717752913 PMC5894917 29630854

[30] Motala MI, Van Wyk JM. Experiences of foreign medical graduates (FMGs), international medical graduates (IMGs) and overseas trained graduates (OTGs) on entering developing or middle income scoping review. BMC 2019; 17: 7. 10.1186/s12960-019-0343-y PMC6341748 30665452

[31] General Medical Council. Welcome to UK Practice. GMC, 2020 (https://www.gmc-uk.org/about/what-we-do-and-why/learning-and-support/workshops-for-doctors/welcome-to-uk-practice/doctors).

[32] Kehoe A, Rothwell C, Hesselgreaves H, Carter M, Illing J. Evaluation of GMC Welcome to UK Practice. Newcastle University, 2019.

[33] Academy of Medical Royal Colleges. Medical Training Initiative. AOMRC, 2020 (https://www.aomrc.org.uk/medical-training-initiative/).

[34] Academy of Medical Royal Colleges. Medical Training Initiative: Relocation Guide. AOMRC, 2018 (http://www.aomrc.org.uk/wp-content/uploads/2018/05/MTI_Relocation_Guide_MAY2018-v5-wr.pdf).

[35] Woolf K. Ethnicity and academic performance in UK trained doctors and medical students: systematic review and meta-analysis. BMJ 2011; 342. Available from: 10.1136/bmj.d901. PMC3050989 21385802

[36] British Medical Association. International Doctors. BMA, 2020 (https://www.bma.org.uk/advice-and-support/international-doctors?utm_source=The%20British%20Medical%20Association&utm_medium=email&utm_campaign=11756811_IMG%20NEWSLETTER%20240820&utm_content=International%20doctors%20resources&dm_i=JVX,6ZZM3,236KBR,S7CO6,1).

[37] Linton S. Taking the difference out of attainment. BMJ 2020; 368. Available from: 10.1136/bmj.m438. 32051115

[38] Woolf K, Viney R, Rich A, Jayaweera H, Griffin A. Organisational perspectives on addressing differential attainment in postgraduate medical education: a qualitative study in the UK. BMJ Open 2018; 8. Available from: 10.1136/bmjopen-2017-021314. PMC5855204 29525774

[39] Greenberg N, Brooks S, Wessely S, Tracy D. How might the NHS protect the mental health of health-care workers after the COVID-19 crisis? Lancet Psychiatry 2020; 7(9): 733–4. 32473664 10.1016/S2215-0366(20)30224-8 PMC7255732

[40] BMJ. *Racism in Medicine*. *BMJ*, 2020 (https://www.bmj.com/racism-in-medicine).

